# Evaluating the Predictive Value of Clinical Factors for Pembrolizumab Efficacy and Safety in Advanced NSCLC with High PD-L1 Expression (TPS ≥ 50%)

**DOI:** 10.3390/jcm14176200

**Published:** 2025-09-02

**Authors:** Fedja Djordjevic, Katarina Ljujic, Nemanja Stanic, Neda Nikolic, Ivan Markovic, Jelena Spasic

**Affiliations:** 1 Clinic for Medical Oncology, Institute for Oncology and Radiology of Serbia, Pasterova 14, 11000 Belgrade, Serbia; katarinaljujic777@gmail.com (K.L.); nemanja.s.stanic@gmail.com (N.S.); neda.nikolic@ncrc.ac.rs (N.N.); 2 Clinic for Surgical Oncology, Institute for Oncology and Radiology of Serbia, Pasterova 14, 11000 Belgrade, Serbia; ivanmarkovic66@yahoo.com; 3 Faculty of Medicine, University of Belgrade, 11000 Belgrade, Serbia

**Keywords:** non-small cell lung cancer, advanced disease, PD-L1 expression, immunotherapy, pembrolizumab, prognostic factors, real world

## Abstract

**Background**: Single-agent pembrolizumab represents a standard of care in the first-line treatment of patients with metastatic non-small cell lung cancer (mNSCLC) with a programmed death-ligand 1 (PD-L1) tumor proportion score (TPS) of ≥50%. Real-world evidence is of increasing importance in oncology, as clinical trial inclusion criteria may not be truly reflective of the patient population seen in daily clinical practice. **Methods**: We performed a prospective–retrospective single-center study including 121 patients who received pembrolizumab as a first-line therapy for mNSCLC with a PD-L1 TPS ≥ 50%. Our aims were to make a comparison with published clinical trial results by assessing the efficacy and safety of pembrolizumab monotherapy in our population. We collected patient demographics, clinical characteristics of the disease, and treatment outcomes, including efficacy and safety. **Results**: A total of 121 patients were included, with a median follow-up of 40.77 months. The median progression-free survival in the real world (rwPFS) was 20.73 months (95% CI 12.24–29.22), and the median overall survival (OS) was 29.30 months (95% CI 16.57–42.04). Immune-mediated adverse events (irAEs) occurred in 42% of patients, with serious events (grade 3 or more) occurring in 12%. ECOG PS 2, male gender, squamous histology, pleural and visceral metastases, and treatment with corticosteroids prior to initiation of pembrolizumab were found to be negative predictors for overall survival, while the occurrence of irAEs was the predictor of longer survival. **Conclusions**: This study provides further real-world insights into the efficacy of pembrolizumab in a heterogeneous patient population with advanced NSCLC in a single center in Serbia. It also confirmed the value of good ECOS PS and the occurrence of irAEs as predictors of favorable outcomes.

## 1. Introduction

Lung cancer (LC) remains a global public health problem despite efforts aimed at reducing and stopping the consumption of tobacco products, early detection, the development of minimally invasive diagnostics, and new therapeutic modalities. In 2020, according to Globocan data, 2,206,771 people were diagnosed with lung cancer, and 1,796,144 died from the disease [1]. Five-year survival of patients suffering from all types of lung cancer is very low (19%), despite the introduction of targeted and immunotherapy [1]. The standard first-line treatment for a patient with locally advanced NSCLC without actionable genomic alterations (AGA), according to the guidelines of all oncology associations, is immunotherapy, delivered as a monotherapy or in combination with platinum-based chemotherapy, depending on programmed death-ligand 1 (PD-L1) expression [2,3]. One of these studies is KEYNOTE-024 (KN024), which established single-agent pembrolizumab for the treatment of advanced NSCLC with PD-L1 expression ≥ 50%, demonstrating significantly longer progression-free (PFS) and overall survival (OS) compared with the investigator’s choice of platinum-based chemotherapy [4,5,6].

Randomized clinical trials are the cornerstone for building medical knowledge [7]. In a post–marketing setting, evaluation of the efficacy and safety of novel therapies through an analysis of clinical experience is now well recognized. This use of real-world data (RWD) is integral to understanding treatment effectiveness, utilization patterns, and outcomes of new treatments outside of the controlled trial setting. It is very important to recognize real patient populations, as clinical trial inclusion criteria may not be truly reflective, and this represents the highest value of real-world evidence.

Serbia is a European country with a very high burden of lung cancer. The incidence has increased over the past three decades, with a standardized incidence rate of 22.4 and 57 in females and males, respectively. In 2019, 6863 new LC cases were diagnosed, comprising 16.2% of all newly diagnosed cancers, making LC the most commonly diagnosed malignant disease [8]. Since 2020, pembrolizumab has been reimbursed as a first-line monotherapy for patients with metastatic NSCLC and PD-L1 expression ≥ 50%.

We performed a single-center prospective–retrospective analysis of a group of patients with metastatic NSCLC (mNSCLC) treated with pembrolizumab monotherapy in the first line, in order to assess the efficacy and safety of the drug in routine clinical practice. The aim of our study was to assess the disease patterns in our cohort of patients and their outcomes, with deeper insight into the clinical characteristics that could be used as clinical prognostic factors.

## 2. Materials and Methods

### 2.1. Patient and Tumor Characteristics

Consecutive patients presenting with mNSCLC, with PD-L1 expression ≥ 50% and without AGA which are currently being tested for in Serbia (EGFR and ALK), who started first line treatment pembrolizumab monotherapy between May 2020 and 31 December 2024. were evaluated. All patients underwent routine PD-L1 testing performed on formalin-fixed paraffin-embedded histology or cytology samples using PD-L1 monoclonal antibodies (22C3 clone by DAKO, Glostrup, Denmark) prior to initiating the first line of treatment. EGFR mutation testing was performed by qPCR (Cobas^®^, Roche Diagnostics Roche Diagnostics, Rotkreuz, Switzerland) and dPCR (QuantStudio Absolute Q Digital PCR System, Thermo Fisher ScientificThermo Fisher Scientific, Waltham, MA, USA), and ALK rearrangements by Ventana anti-ALK (D5F3) (Rabbit Monoclonal Primary Antibody, Roche Diagnostics GmbH, Mannheim, Germany) prior to the first-line treatment initiation.

Patients were identified using hospital electronic records, and a subsequent review of individual charts was performed. Data on patients’ baseline demographics were collected as follows: age at diagnosis of stage IV NSCLC, sex, European Cooperative Oncology Group performance status (ECOG PS), and smoking history. Smoking status was categorized as never (i.e., never smoked), current (i.e., still smoking or stopped within 1 year of diagnosis), and former (i.e., other smoking status). Other parameters that were recorded and followed were as follows: date of diagnosis, histology, PD-L1 tumor proportion score, initial staging (cTNM per AJCC TNM8 staging system), site of metastases, treatment with corticosteroids within one month prior to and during immunotherapy, radiotherapy during pembrolizumab, adverse events, and post-progression type of treatment provided. PD-L1 expression was determined via immunohistochemistry, as the percentage of membranous-stained positive tumor cells using the 22C3 pharm Dx assay, and is presented in subgroups of 10% (Table 1).

### 2.2. Treatment and Follow-Up

Pembrolizumab monotherapy was administered at a fixed dose of 200 mg continuously every three weeks until the occurrence of progressive disease, death from any cause, unacceptable toxicity, or patient preference. The administration of up to 10 mg of a prednisolone equivalent at the start of pembrolizumab therapy was defined as a threshold to discriminate patients with steroid treatment versus those without [9]. The primary survival endpoint was the overall survival (OS), defined as the time in months from the diagnosis to death from any cause or date of last contact. Real-world progression-free survival (rwPFS) was also followed, and defined as the time from the start of pembrolizumab to the confirmed progression of disease. Other efficacy endpoints included the response rate (RR) and disease control rate (DCR), defined as the percentage of patients whose disease did not progress (meaning that they achieved a complete response, partial response, or stable disease). The therapeutic response was assessed according to Response Evaluation Criteria In Solid Tumors 1.1 (RECIST 1.1) [10] by the investigators. The treatment given after progression on pembrolizumab was noted. Safety assessments were performed according to the NCI Common Terminology Criteria for Adverse Events (CTCAE), version 5.0 [11]. Approval for the study was obtained from the Ethics Board of the Institute for Oncology and Radiology of Serbia (approval number 01-1/2024/678), dated 1 March 2024.

### 2.3. Statistical Methods

Descriptive statistics were calculated for the baseline demographic, clinical features, and treatment outcomes. Graphical and mathematical methods tested the normality of distribution (Normal Q-Q Plot, Shapiro–Wilk test). Continuous variables were presented as means with standard deviations or medians with 25th–75th percentiles, as appropriate. Categorical variables were presented as counts and percentages. The data cut-off date was the 31st of May 2025. Curves of probabilities for OS were constructed using the Kaplan–Meier product-limit method; the median of survival analysis with corresponding 95% CI was used for the description, and the log-rank test was utilized to test for differences between curves. The univariate Cox proportional hazard regression model was used to determine a hazard ratio (HR) corrected for possible confounding factors. Multivariable Cox regression and binomial logistic regression were used to determine the effect of multiple predictor variables on OS and the objective response rate (ORR). The level of significance was set at 0.05. Statistical analysis was performed using the IBM SPSS 21 (IBM, Chicago, IL, USA, 2012) package [12].

## 3. Results

### 3.1. Patients’ Baseline Characteristics

We included 121 consecutive patients with metastatic NSCLC, with a PD-L1 TPS ≥ 50% and without EGFR and ALK alterations. Table 1 shows the baseline characteristics of the whole cohort. The median age of patients was 67 years, with a range from 20 to 87 years, while over 70% of patients were between 60 and 79 years old at the time of diagnosis. The cohort included more male than female patients (57% vs. 43%). Regarding smoking status, 53% were never smokers, 36% were former smokers, and 11% were active smokers at the start of treatment. Most patients had ECOG PS 1 (63%), while 15% had ECOG PS 0, and 22% had ECOG PS 2. Adenocarcinoma was the most common histological subtype, present in 70% of patients, followed by squamous cell carcinoma in 25%, and not otherwise specified (NOS) NSCLC in 5%. Expression levels of PD-L1 were 50–60% in 34 patients, 61–70% in 25, 71–80% in 26, 81–90% in 26, and 91–100% in 10 patients. Prior to immunotherapy initiation, 23% of patients were treated with corticosteroids, mostly to treat symptoms of brain or bone metastases.

The number and location of metastatic sites are presented in Table 2. Most frequently, patients presented with metastases in two metastatic sites. The most frequent metastatic sites were lymph nodes, contralateral lung, pleura, and brain.

### 3.2. Efficacy

As of 31 May 2025, the median follow-up was 40.77 (CI 95%; **34.39–4.3**) months, and 30 (25%) of the patients were still receiving pembrolizumab treatment (54 patients for longer than 12 months). The median number of treatment cycles was 8 (1–56). Radiotherapy during pembrolizumab treatment was used in 6% of patients to treat painful bone metastases or sites of oligoprogressive disease, with palliative intent. The best response was CR in only 0.8% of patients, PR in 32%, and SD in 53%, with an objective response rate (ORR) of 33%, and a disease control rate (DCR) of 75.8%. At data cut-off, progressive disease was confirmed in 48% of patients, while 52% had died, suggesting death without confirmed disease progression in some of them. A quarter of the patients received further treatment after progression, such as chemotherapy 14%, followed by radiotherapy in 7.4% of cases, and chemo-radiotherapy in 5% (Table 3).

**Figure 1:**
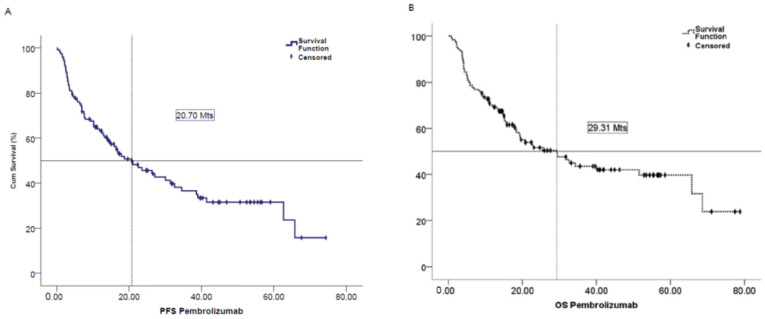
Progression-free survival (**A**) and Overall survival (**B**).

The median real-world progression-free survival (mrwPFS) was 20.73 months (95% CI 12.24–29.22), and is shown in Figure 1A. The mOS was 29.30 months (95% CI 16.57–42.04), and is shown in Figure 1B. Kaplan–Meier estimates of OS at 12, 24, and 48 months were 90.1%, 80.2%, and 67%, respectively.

### 3.3. Impact of Clinical Characteristics on the Efficacy of Pembrolizumab

**Figure 2:**
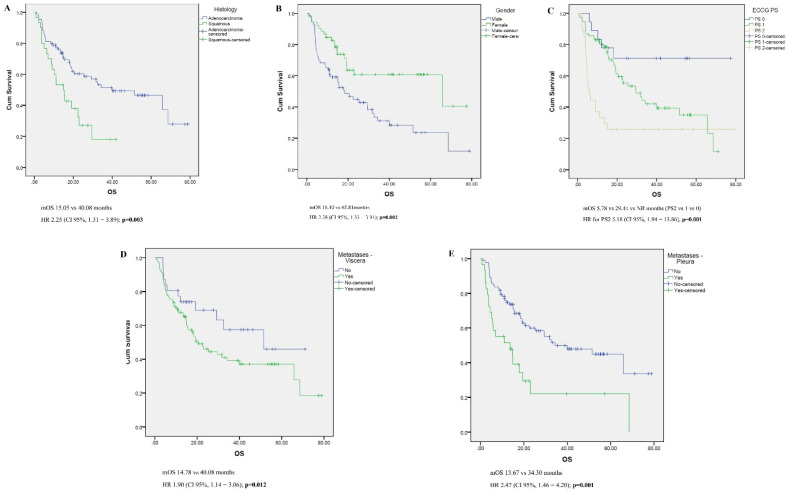
Kaplan–Maier survival curves showing the effect on OS of the following characteristics (univariate analysis): histology (**A**), sex (**B**), ECOG PS (**C**), the presence of visceral metastases (**D**), and the presence of pleural metastases (**E**).

Differences in the overall survival according to patient and tumor characteristics are presented in Table 4 and Figure 2. Male gender, ECOG PS 2, squamous cell histology, the presence of pleural and visceral metastasis, and corticosteroid use at the beginning of treatment were all linked to worse outcomes in the univariate analysis. However, when multivariate analysis was performed on these five variables, only ECOG PS and the presence of pleural metastases remained significant predictors for OS. The logistic regression model was statistically significant, χ2(5) = 24.345, *p* < 0.001. The model explained 25.0% (Nagelkerke R2) of the variance in ORR and correctly classified 73.6% of cases. Sensitivity was 82.7%, specificity was 55.0%, positive predictive value was 78.8%, and negative predictive value was 61.1%. Of the five predictor variables, only the following two were statistically significant: ECOG PS (HR 3.352 (1.506–7.460), *p* = 0.003) and pleural metastases (HR 6.864 (1.749–26.942), *p =* 0.006).

Interestingly, there were no differences in OS regarding age, smoking status, the presence of brain metastases, and the level of PD-L1 expression, although we did detect a trend towards a longer OS in patients under the age of 70.

### 3.4. Safety

Immune-mediated adverse events (irAEs) occurred in 52 patients (42%), with grades of 1–5. The incidence of serious irAEs (grades 3–5) was 12%; two treatment-related fatal AEs occurred. Most of the adverse events were mild (grades 1 and 2). Skin changes in G2 were the most common adverse events, followed by arthritis. More than two different irAEs occurred in 10 patients (8.3%), with 4 of them experiencing three different irAEs, and 7 of them discontinuing treatment due to AEs. Overall, treatment with pembrolizumab was discontinued due to irAEs in 21 (18%) patients (Table 5).

**Figure 3:**
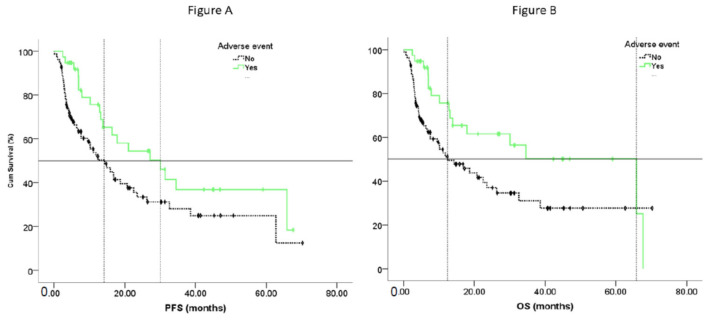
Association between irAEs and survival, PFS (**A**) and OS (**B**).

We found a significant association between the occurrence of irAEs and the efficacy of pembrolizumab. In patients with irAEs, the median rwPFS was 31.38 months (CI 95%; 20.70–40.05), and 15.87 (CI 95%; 8.858–22.879) in patients without, *p* = 0.030; the median OS in the group of patients with AEs was 65.81 (CI 95%; 21.09–110.52) months, and 19.25 (CI 95%; 9.29–29.21) in those without, *p* 0.019; HR 1.95 (CI 95% 1.10–3.45) (Figure 3).

## 4. Discussion

This study provides efficacy and safety data concerning pembrolizumab monotherapy in advanced or recurrent PD-L1-high NSCLC in a real-world setting. We followed 121 patients with stage IV NSCLC without EGFR and ALK alterations, with high PD-L1 TPS levels (≥50%), who were treated with pembrolizumab in a single institution. In this group of patients, the efficacy of pembrolizumab corresponds well with data from the registrational KN024 study and, in fact, was slightly better. With a median follow-up of 40.77 months, the median OS was 29.30 months (whereas the mOS in KN024 was 26.3 months), and the survival rates at 12, 24, and 48 months are also higher than in KN024 [6]. This is an interesting finding, considering the differences in the study populations, with our group of patients having a higher percentage of characteristics that are historically regarded as unfavorable, but, in our opinion, are more reflective of real-world patients, at least in our region. We had as many as 22% of patients with ECOG PS 2 (excluded in KN024), more patients with squamous cell carcinoma (24% vs. 18.8%), our patients were slightly older (median age 67 vs. 64.5), and with more than double the percentage of patients having brain metastasis at baseline (21.5% vs. 11.7%). Also, most of our patients were non-smokers and former smokers (53% and 36%, respectively), while in KN024, patients were mostly former (74.7%) and current smokers (22.1%). This is somewhat surprising, and not reflective of local conditions in Serbia, which is still a country where consumption of tobacco products is among the highest in Europe, and where there are still no significant restrictions on smoking in public areas. The male-to-female ratio did not differ, but the female gender was a strong predictor of good outcomes in our study, which is also different than KN024.

With five years of follow-up in KN024, the 12, 24, and 48-month OS rates for patients in the pembrolizumab arm were 70.3%, 51.5%, and 35.8%, respectively. The 12, 24, and 48-month OS rates for patients in our study were 90.1%, 80.2%, and 67%, respectively. Several other observational studies have been published recently that evaluated outcomes of first-line pembrolizumab in the same patient population. As expected, there was some variability in patient populations and survival. The reported median OS varied between 17.2 and 24.3 months [13,14,15,16], which is comparable to our results.

In this analysis, several factors proved to be prognostic for shorter survival in a univariate analysis (male gender, ECOG PS ≥ 2, squamous histology, pleural metastasis, visceral metastasis other than the lung, and corticosteroid use prior to pembrolizumab initiation). However, when analyzed in a multivariate regression analysis, only ECOG PS and the presence of pleural metastases remained prognostic factors.

In patients presenting with advanced NSCLC, ECOG PS2 is expected in around 18% of patients [17]. These patients were not included in most of the registration trials, so real-world results are of great importance. In RWD studies, patients with PS2 represented about a quarter to a third of all patients, with 22% in our study and up to 34% in others [16]. A retrospective study carried out by Middleton et al. [17] showed a promising survival of 9.8 months in patients with PS2 treated with pembrolizumab. In our group, patients with ECOG PS 2 had only 5 months of survival, which is similar to results from other RWD studies [16]. Poor ECOG performance status has been proven to be an independent predictor of shorter survival, regardless of treatment. Nevertheless, the evidence for making treatment decisions about using immunotherapy in this group is still scant. There is data from the recent IPSOS phase III study [18], which suggest the benefits of single-agent immunotherapy in patients with mNSCLC who are older, frail, have substantial comorbidities, and have ECOG PS2 or 3. More data are needed to fully prove whether PS2 is only a prognostic marker or both a prognostic and a predictive marker for immunotherapy efficacy.

In our group of patients, pleural, brain, liver, and bone metastases were recorded in 24%, 21.5%, 12%, and 15% of patients, respectively, while the majority of patients had lung metastases (52%). Pleural metastasis proved to be a prognostic factor for survival, as well as visceral metastasis other than the lung. This finding is in concordance with the results of KN024 and other RWD results [6,13,14,15].

Brain metastases (BM) are expected in 30% to 40% of patients with mNSCLC, and have been associated with a poor prognosis and quality of life [7]. Observational studies suggest that the survival benefit from pembrolizumab-based therapy may not be inferior for patients with BM, as well as subgroup analyses from randomized studies [19,20]. In our cohort, BM at baseline were not connected with worse outcomes. Patients with BM at baseline compared to patients without BM did have a shorter OS, but the difference in the median OS was not statistically significant.

The use of corticosteroids prior to immunotherapy was also recognized as a negative prognostic factor. Corticosteroids were introduced to reduce the symptom burden related to the disease, mainly in the treatment of CNS and bone metastasis. Other RWD studies, as well as randomized trials, suggest the same result [13].

The level of expression of PD-L1 did not have an effect on OS, even in those patients with a PD-L1 expression over 90%, despite data from the literature, which suggest a positive correlation between high expression of PD-L1 and OS [21].

After progression on first-line pembrolizumab monotherapy, a high percentage of our patients did not receive any further oncologic treatment, with only 25% being fit enough for subsequent treatment (chemotherapy 14%, radiotherapy 7.4%, and chemo-radiotherapy 5%). Looking back at the results from KN024, more than half of the patients in the pembrolizumab arm received subsequent anticancer treatments. On the other hand, in the analysis from Cortelini [13], also retrospective in nature, 35.5% of included patients received second-line systemic treatment. We feel that this is also a reflection of real-world patients we see in everyday clinical practice, and again, an important difference from the controlled environment of clinical trials.

In this study, the profile of irAEs was similar to previous reports, and no unexpected irAEs were observed. IrAEs occurred in 52 patients (42%) at grades 1–5. The incidence of serious irAEs (grades 3–5) was 12%, with two treatment-related fatal AEs (myocarditis and hypophysitis). Skin changes were the most common side effects, followed by arthritis. The occurrence of AEs had a prognostic value, i.e., the patients in whom AEs occurred had longer survival times. The DCR was 91.2% versus 70% in those without documented AEs, which was statistically significant and resulted in differences in PFS and OS between these groups. The median PFS in the group with AEs was 30 months, and 14.3 months in the group without (*p* = 0.034). The median OS in the group of patients with AEs was 65.8 months, and 12.45 months in the group without, HR 1.93 (CI 95% 1.09–3.41; *p* = 0.022). Again, these findings are in concordance with randomized trials of immunotherapy, as well as many reported RWD studies. In fact, Cortelini et al. [13] and Wu and colleagues [22] found that the occurrence of multiple-site irAEs in a single patient was associated with longer survival than the occurrence of a single-site irAE. In our group, multiple-site irAEs occurred in only ten patients, so this number is too small for such an analysis.

Another study also conducted in Serbia [23] found a total of 61 immune-related adverse events (irAEs) observed in 47 patients (20.8%), which is a slightly lower percentage than expected and also lower than we observed in our group of patients. This goes to show the heterogeneity of data in real-world settings, which is influenced by the ways of collecting data and the follow-up of patients.

The results of this study should be considered in the context of the strengths and limitations of the study design. This study has several strengths, including a large patient population and a long follow-up period to record outcomes in the real-world clinical setting in a previously under-represented population, as not many clinical trials were conducted in Serbia in recent years. Our patient population was heterogeneous, with a high percentage of patients having unfavorable characteristics, which is highly reflective of routine clinical practice.

We have to acknowledge a major limitation of this study, which is also reflective of routine clinical practice in Serbia—a lack of comprehensive genomic profiling of these tumors. Due to reimbursement restrictions, only EGFR and ALK alterations are routinely tested, so it is reasonable to assume that there were some patients in this study whose tumors had some other targetable alterations. Missing data for other key variables, such as TMB, KRAS mutations, and co-mutational status (e.g., TP53), prevented us from conducting a powered analysis that includes these variables, and we know that increasing TMB levels are associated with immune cell infiltration and an inflammatory T-cell-mediated response, resulting in increased sensitivity to PD-1/PD-L1 blockade in NSCLC across PD-L1 expression subgroups, while KRAS mutations confer worse outcomes on immunotherapy [24,25]. Further studies are in progress and being planned, which will include several of these variables.

With the emergence of new classes of drugs (molecularly targeted and immunotherapy) in the therapeutic approach of treating a patient diagnosed with advanced NSCLC, treatment outcomes have changed significantly. Survival was prolonged, as was the quality of life of the patients. There are clear differences in the effectiveness of immunotherapy between patients, as well as in the toxicity shown; however, it is still not entirely clear why immunotherapy is more effective in some patients, or why serious side effects occur more often than others. Therefore, it is crucial to continue with studies such as ours, all with the goal of providing a more personalized approach to treatment.

## 5. Conclusions

This study represents an important and large comprehensive analysis of Serbian patients with metastatic PD-L1-high NSCLC, albeit in a single institution. Here, pembrolizumab provided the expected clinical benefit and toxicity profile. Our data indicated a comparable survival outcome for pembrolizumab treatment with previously published data. While characteristics that showed a significant effect on the efficacy and toxicity of pembrolizumab are not new and unknown, this study adds to the growing amount of real-world evidence in treating this often challenging patient population. By providing granular real-world evidence, we aimed to bridge the gap between trial data and clinical practice, emphasizing the importance of patient selection. Further studies, with a larger patient population and a more comprehensive evaluation of patient and tumor-related factors, are needed for a better understanding of immunotherapy outcomes and the prediction of response.

## Figures and Tables

**Table 1:** Baseline patient characteristics.

Parameters		Number (%)
**Sex**	Female	52 (43)
	Male	69 (57)
**Age**	≤39 years	4 (3)
	40–49	6 (5)
	50–59	22 (18)
	60–69	41 (34)
	70–79	45 (37)
	≥80	3 (2)
**ECOG PS**	PS 0	18 (15)
	PS 1	76 (63)
	PS 2	27 (22)
**Histology**	Adenocarcinoma	85 (70)
	Squamous	30 (25)
	NOS	6 (5)
**Smoking Status**	Former	44 (36)
	Never-smoker	64 (53)
	Current smoker	13 (11)
**Corticosteroid use**	No	93 (77)
	Yes	28 (23)
**PD-L1 expression**	50–60%	34 (28)
	61–70%	25 (21)
	71–80%	26 (21.5)
	81–90%	26 (21.5)
	≥91%	10 (8)

**Table 2:** Metastatic sites.

Localization	Category	Number (%)
N° of localizations	One	26 (21.5)
	2	51 (42)
	3	32 (26)
	4	9 (7)
	≥5	3 (2.5)
Brain		26 (21.5)
Lung, contralateral		63 (52)
Lymph nodes		96 (79)
Liver		15 (12)
Bone		19 (15)
Pleura		29 (24)
Adrenal		17 (14)
Other		14 (12)

**Table 3:** Efficacy outcomes.

Parameters	Category	Number (%)
N° of Pembrolizumab cycles	1–8	66 (54.5)
	9–16	29 (24)
	≥17	26 (21.5)
Best objective response	CR	1 (0.8)
	PR	39 (32)
	SD	64 (53)
	PD	16 (13)
Progression confirmed		58 (48)
Treatment after the progression	CHT	17 (14)
	RT	9 (7)
	CRT	6 (5)
	BSC	89 (74)
Death		63 (52)

Abbreviations: CR—complete response; SD—stable disease; PR—partial response; PD—progressive disease; CHT—chemotherapy; RT—radiation therapy; BSC—best supportive care.

**Table 4:** Patient and disease characteristics and influence on overall survival.

Category		Median	CI 95%	Log Rank	HR (CI 95%)
Sex	Male	18.40	10.08–26.72	0.002	2.28 (1.33–3.91)
	Female	65.81	2.14–129.47		
Age	<70 years	31.74	11.85–62.62	0.072	NS
	≥70 years	18.40	4.14–32.66		
ECOG PS	PS 0	NR	/	0.001	* PS1 2.33 (0.92–5.91) PS2 5.18 (1.94–13.86)
	PS 1	29.44	18.13–40.70		
	PS 2	5.78	3.55–8.01		
Histopathology	Adenocarcinoma	40.08	17.11–63.05	0.003	2.25 (1.31–3.89)
	Squamous	15.05	8.97–21.12		
Metastatic localizations	One	NR	/	0.449	NS
	Two	31.74	15.98–47.49		
	Three	18.17	12.49–23.84		
	4 or more	5.19	1.01–36.12		
Metastases—CNS	No	22.505	12.37–32.64	0.406	NS
	Yes	51.483	36.16–66.80		
Metastases—Pleural	No	34.300	9.21–59.39	0.001	2.47 (1.46–4.20)
	Yes	13.667	1.47–25.87		
Metastases—Visceral **	No	40.082	13.97–66.19	0.012	1.90 (1.14–3.06)
	Yes	14.784	5.33–24.24		
PD-L1 expression	50–60	19.417	9.49–29.34	0.525	NS
	61–70	29.437	0.17–58.70		
	71–80	18.398	13.46–23.34		
	81–90	32.263	6.60–58.59		
	91–100	NR	/		
Corticosteroid use	No	32.591	9.26–55.92	0.001	2.41 (1.39–4.17)
	Yes	9.101	0.00–19.59		

NR—Not Reached; NS—Not significant; * Multivariable Cox Regression was used for calculation; ** Metastases in viscera other than the lung, excluding lymph nodes and bones.

**Table 5:** Side effects.

Grade	1	2	3	4	5	Total
Pneumonitis	0	1	1	0	0	2
Mucositis	0	2	3	0	0	5
Thyrotoxicity	4	2	0	0	0	6
Hepatitis	0	2	2	2	0	6
Skin changes	5	7	1	1	0	14
Hypophysitis	0	0	1	0	1	2
Myositis	0	1	0	0	1	2
Arthritis	1	7	1	0	0	9
Colitis	0	5	1	0	0	6
**Total**	10	27	10	3	2	52

## Data Availability

Data is contained within the article. The original contributions presented in this study are included in the article. Further inquiries can be directed to the corresponding authors.

## Abbreviations

The following abbreviations are used in this manuscript:

NSCLC	non-small cell lung cancer
mNSCLC	metastatic non-small cell lung cancer
PD-L1	programmed death-ligand 1
TPS	tumor proportion score
OS	overall survival
I-rAE	immune-mediated adverse events
EGFR	epidermal growth factor receptor (EGFR)
ALK	anaplastic lymphoma kinase
rwPFS	real-world progression-free survival
RWD	real-world data
LC	lung cancer
RECIST 1.1	response evaluation criteria in solid tumors
CTCAE 5.0	common terminology criteria for adverse events
HR	hazard Ratio
ORR	objective response rate
PR	partial response
SD	Stable disease
PD	progressive disease
CR	complete response
CHT	chemotherapy
RT	radiation therapy
BSC	best supportive care
NR	not reached
NS	not significant
BM	brain metastases
